# Predawn leaf water potential of grapevines is not necessarily a good proxy for soil moisture

**DOI:** 10.1186/s12870-023-04378-6

**Published:** 2023-07-25

**Authors:** Thomas Groenveld, Charles Obiero, Yingxue Yu, Markus Flury, Markus Keller

**Affiliations:** 1 Department of Viticulture and Enology, Irrigated Agriculture Research and Extension Center, Washington State University, Prosser, WA USA; 2 Present Address: Central and Northern Arava Research and Development Center, Hatzeva, Israel; 3 Department of Crop and Soil Sciences, Puyallup Research & Extension Center, Washington State University, Puyallup, WA USA; 4 Department of Crop and Soil Sciences, Washington State University, WA Pullman, USA

**Keywords:** Soil water potential, Model, Nighttime transpiration, Disequilibrium, *Vitis*

## Abstract

**Background:**

In plant water relations research, predawn leaf water potential (Ψ_pd_) is often used as a proxy for soil water potential (Ψ_soil_), without testing the underlying assumptions that nighttime transpiration is negligible and that enough time has passed for a hydrostatic equilibrium to be established. The goal of this research was to test the assumption Ψ_pd_ = Ψ_soil_ for field-grown grapevines.

**Results:**

A field trial was conducted with 30 different cultivars of wine grapes grown in a single vineyard in arid southeastern Washington, USA, for two years. The Ψ_pd_ and the volumetric soil water content (θ_v_) under each sampled plant were measured multiple times during several dry-down cycles. The results show that in wet soil (Ψ_soil_ >  − 0.14 MPa or relative extractable water content, θ_e_ > 0.36), Ψ_pd_ was significantly lower than Ψ_soil_ for all 30 cultivars. Under dry soil conditions (Ψ_soil_ <  − 0.14 MPa or θ_e_ < 0.36) Ψ_pd_ lined up better with Ψ_soil_. There were differences between cultivars, but these were not consistent over the years.

**Conclusion:**

These results suggest that for wet soils Ψ_pd_ of grapevines cannot be used as a proxy for Ψ_soil_, while the Ψ_pd_ = Ψ_soil_ assumption may hold for dry soils.

## Background

Water scarcity and the increasing frequency and severity of drought episodes are driving horticultural and ecological research to study water stress tolerance and avoidance in various plant species [1, 2]. A key challenge when studying water relations in plants is integrating many parameters, such as stomatal conductance (g_s_), whole-plant (i.e., root to leaf) hydraulic conductance (K), leaf water potential (Ψ_leaf_), atmospheric vapor pressure deficit (VPD) and soil water potential (Ψ_soil_), which vary in space and time during water stress establishment. Soil water content is difficult to quantify due to heterogeneity of the water distribution in the soil. By contrast, Ψ_leaf_ measurements do not have this limitation because the plant is affected by the Ψ_soil_ across its entire root system and is thought to equilibrate at night to the highest Ψ_soil_ according to the root density distribution [3]. Mechanistic models describing water flow in the soil–plant–atmosphere continuum are usually analyzed by means of Ohm’s law [4]. Under the assumption that stomatal closure at night prohibits transpiration and that there was ample time for hydraulic equilibrium to be established, predawn leaf water potential (Ψ_pd_) has been proposed as a proxy for Ψ_soil_ [5]. Many studies and models exploring plant water relations are based on Ψ_pd_ being a proxy for Ψ_soil_ without explicitly testing the underlying assumptions [6–10]; “Ψ_pd_ = Ψ_soil_” has become a ‘rule of thumb’.

Numerous authors have published data showing a disequilibrium between Ψ_pd_ and Ψ_soil_. Donovan et al. [11] referenced 32 publications that tested the equilibrium between Ψ_pd_ and Ψ_soil_, and approximately half of these papers reported the Ψ_pd_ to be at least 0.5 MPa lower than Ψ_soil_. In their own data, 15 of the 21 species they grew in greenhouses under well-watered conditions showed a disequilibrium, which they found to be mostly due to nighttime transpiration and, for some species, due to accumulation of apoplastic solutes in intercellular leaf spaces. The magnitude of the disequilibrium varied with species; desert shrubs showed the highest predawn disequilibrium and temperate species the lowest.

In regard to grapevine (*Vitis vinifera* L.), which has been used as a model species for water relations in perennial plants, some authors have published significant correlations between Ψ_pd_ and Ψ_soil_, but their data show there is a considerable difference in the wetter soil range [12], or that the slope of the linear regression is greater than 1 MPa MPa^−1^ [13]. Despite numerous publications that indicate there is a disequilibrium between Ψ_pd_ and Ψ_soil_ and possible causes for this, models that are based on the equilibrium assumption continue to be published, including models for irrigation scheduling and models that divide plants, including grape cultivars, into different levels of isohydricity [6, 7, 9]. A recurring case is the use of the evaporative flux method (EFM), which is a common approach to model water flow in the soil–plant–atmosphere continuum and to estimate K (mmol m^−2^ s^−1^ MPa^−1^):

1 $$K=\frac{E}{{\Psi }_{soil}-{\Psi }_{leaf}}$$

where E is the transpiration rate per unit leaf area (mmol m^−2^ s^−1^), and Ψ_soil_ is typically replaced by Ψ_pd_. Many authors have relied on this approach to build models that describe the plant water status behavior under drought stress [6, 7, 14], even if actual soil water content measurements are available [15, 16]. Martínez-Vilalta et al. [7] used the Ψ_pd_ = Ψ_soil_ assumption with the EFM to describe how the pressure drop or the water potential difference from soil to plant (ΔΨ = Ψ_soil_ ‒ Ψ_leaf_) progresses as soil moisture is depleted. They tested their model on 102 plant species, demonstrating different behaviors among them. But considering the abundant evidence of Ψ_pd_ to Ψ_soil_ disequilibrium, such model results and the applications based on them could be unreliable. There is a need to quantify the difference between Ψ_soil_ and Ψ_pd_ before comparing different genotypes, both between and within species, for their water stress response. The Ψ_pd_ = Ψ_soil_ assumption in the EFM continues to be used to explain differences in hydraulic behavior between cultivars of the same species [17]. Since intra-species differences in water relations have been reported in wine grapes [1, 18], it is possible that variability in the Ψ_pd_ to Ψ_soil_ disequilibrium also exists among different cultivars and not just among different species. Exploring the magnitude of such variation would enhance the reliability of comparisons of the water stress responses of different cultivars as well. If a consistent difference between Ψ_soil_ and Ψ_pd_ could be found for a particular genotype, then it would be possible to introduce a genotype-specific correction factor for use in modeling approaches based on the EFM.

Given the high economic importance of *V. vinifera*, especially in seasonably dry climates, and the extensive work done to understand water stress adaptability of some of its more than 5000 cultivars [17, 19], our objective was to monitor the behavior of soil water status and predawn plant water status of 30 wine grape cultivars grown side by side during soil dry down from above field capacity to close to the permanent wilting point. We aimed to test two hypotheses: (i) grapevine Ψ_pd_ equilibrates with Ψ_soil_ across the soil moisture spectrum (i.e., Ψ_pd_ = Ψ_soil_); and (ii) if the first hypothesis cannot be confirmed, then the predawn disequilibrium differs among different *V. vinifera* cultivars, making it important to quantify it for each cultivar before comparing different cultivars for their water stress responses.

## Results

### Retention curve

**Fig. 1:**
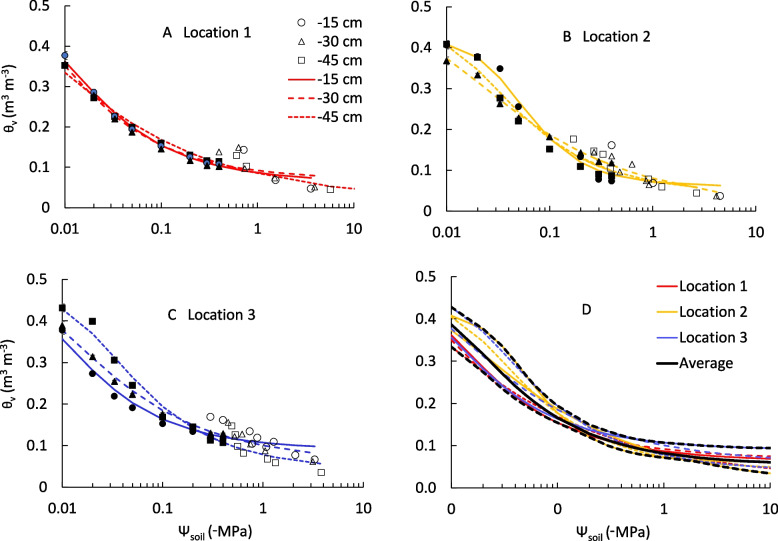
Volumetric soil water content (θ_v_) versus soil water potential (Ψ_soil_) of soil from 3 locations (45 m apart) and 3 depths, plotted with the retention curves. Filled symbols are data points measured using a pressure chamber, non-filled symbols are those measured by means of a dew point potentiometer. The bottom right graph shows all the retention curves together and the average retention curve which was calculated from all data combined, the confidence interval based on the highest and lowest retention curve values for a given θ_v_ is indicated in black dashed lines

Despite the vineyard being categorized as a single soil type on the USDA soil classification [20] and its physically homogenous appearance, the retention curves based on parameters determined for each of the nine soil samples varied considerably (Fig. 1, Table 1). The ANOVA, however, showed that there was no significant effect of sampling location (*p* = 0.80) or depth (*p* = 0.27) on the relation of Ψ_soil_ to volumetric soil water content (θ_v_), and there was no interaction between location and depth (*p* = 0.93). The nine retention curves were thus considered to represent the variability in soil water retention across the field.

**Table 1:** Parameters for the van Genuchten model (Eq. 2) optimized for the retention curve data of 9 soil samples from a vineyard in southeastern Washington and the r^2^ of the optimized model to the data, estimated by means of the RETC code [21]. The average was determined by fitting the retention curve to the pooled data of all samples

*Location*	*Depth (cm)*	*θ*_*r*_* (m*^*3*^* m*^*−3*^*)*	*θ*_*s*_* (m*^*3*^* m*^*−3*^*)*	*α (cm*^*−1*^*)*	*n*	*r*^*2*^
1	15–22	0.063	0.49	0.013	1.591	0.980
1	30–37	0.07	0.51	0.017	1.571	0.976
1	45–52	0.02	0.46	0.025	1.344	0.989
2	15–22	0.06	0.42	0.003	2.047	0.964
2	30–37	0.00	0.46	0.011	1.370	0.989
2	45–52	0.04	0.45	0.005	1.642	0.971
3	15–22	0.09	0.49	0.014	1.632	0.965
3	30–37	0.06	0.49	0.013	1.464	0.987
3	45–52	0.04	0.48	0.005	1.604	0.979
*Average*		*0.05*	*0.47*	*0.008*	*1.601*	*0.938*

The average θ_v_ of the vineyard soil at field capacity (Ψ_soil_ =  − 0.033 MPa) was 0.26 m^3^ m^−3^ (standard deviation, SD = 0.04 m^3^ m^−3^, *n* = 27 pressure plate values), and the average θ_v_ at permanent wilting point (Ψ_soil_ =  − 1.5 MPa) was 0.08 (SD = 0.01) m^3^ m^−3^ (*n* = 6 dew point potentiometer values). The parameters of Eq. 2 fitted to the retention curve data are listed in Table 1. The θ_s_ for the different soil samples varied from 0.421 to 0.507 m^3^ m^−3^, θ_r_ varied from 0 to 0.09 m^3^ m^−3^, α varied from 0.003 to 0.025 cm^−1^, and n varied from 1.344 to 2.047, which indicates variability due to sample location and depth. The parameters called ‘average’ in Table 1 are those of the fit of Eq. 2 to the pooled data set of all soil samples, and these were the parameters used in the subsequent analysis of Ψ_pd_ in relation to Ψ_soil_. The maximal and minimal Ψ_soil_ for each θ_v_ (black dashed lines in the graph showing all the retention curves together in Fig. 1) were used to represent the variability around the average retention curve.

### Soil and leaf water potential

**Fig. 2:**
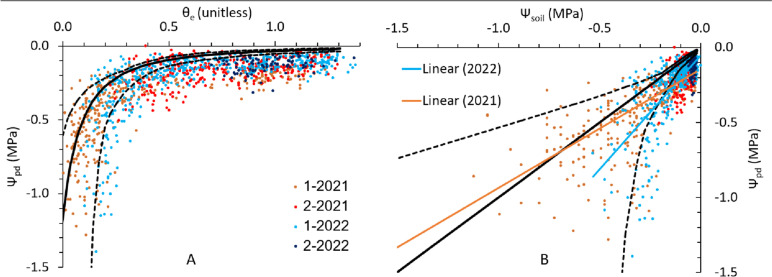
Predawn leaf water potential (Ψ_pd_) of 30 grape cultivars as a function of **A**: the relative extractable soil water (θ_e_, Eq. 4), and **B**: the soil water potential (Ψ_soil_). The legend indicates the number of the dry down cycle and the year in which the measurements were done. The solid line indicates the average retention curve, and the dashed lines indicate the maximal and minimal retention curves. The linear trendlines are shown for the data combined for each season

The Ψ_pd_ data for all 30 grape cultivars and 2 years (*n* = 1215) are presented as a function of the highest relative extractable soil water content (θ_e_) measurement in Fig. 2A, and the same data are plotted as a function of Ψ_soil_ in Fig. 2B. In the wet soil range most Ψ_pd_ values are lower than the Ψ_soil_. At θ_e_ > 0.36 (equivalent to Ψ_soil_ >  − 0.14 MPa or θ_v_ > 0.146 m^3^ m^−3^), 90% of the Ψ_pd_ measurements fall outside the range of potential retention curves. In the dry range Ψ_pd_ is more similar to Ψ_soil_. At θ_e_ < 0.36, 68% of the Ψ_pd_ measurements fall within the range of potential retention curves, and at θ_e_ < 0.14 (equivalent to Ψ_soil_ <  − 0.37 MPa or θ_v_ < 0.105 m^3^ m^−3^) 90% do.

**Fig. 3:**
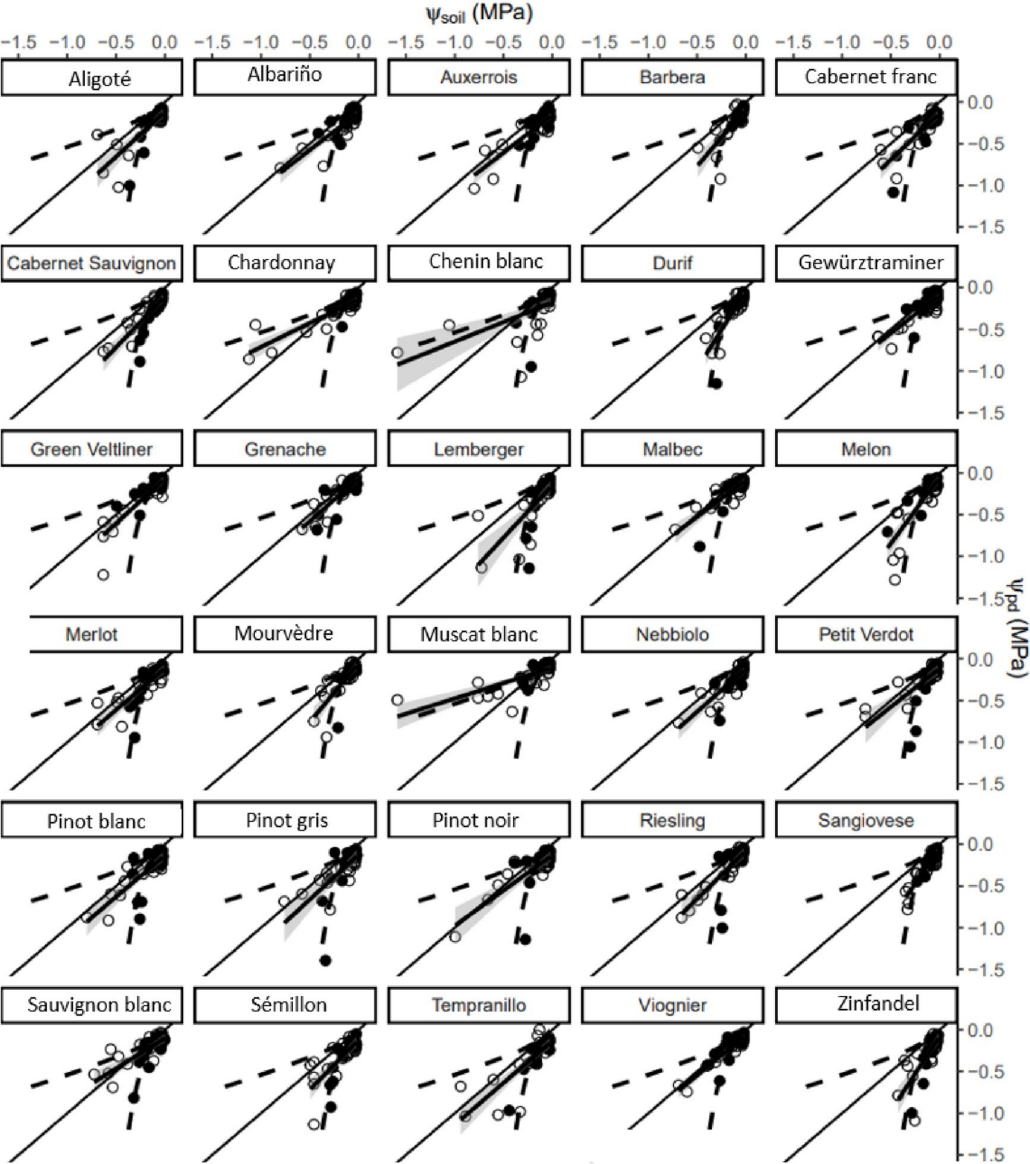
Predawn leaf water potential (Ψ_pd_) of 30 grape cultivars plotted as a function soil water potential (Ψ_soil_) over two years with a linear regression line plotted in black, and grey-shaded bands showing the 95% confidence interval. The empty symbol (○) represents the 2021 data and the filled symbol (●) is 2022. The dashed lines are the minimal and maximal Ψ_soil_ values from retention curves of 9 soil samples shown as the confidence interval in Fig. 1. The slopes and intercepts of the linear models are reported in Table 2

The Ψ_pd_ measurements are plotted as a function of Ψ_soil_ for each of the 30 cultivars in Fig. 3, with the linear regression model for the data of both years combined and displayed with the 95% confidence area. The maximal and minimal potential retention curves are plotted on the same graph, as these represent the measured variability and could represent the retention curve of the soil the plants were grown in, which would shift the data closer to or away from the 1:1 line. The intercept of the linear regression model is negative for all cultivars with an average value of − 0.1 (SD 0.04) MPa (Fig. 2B) and ranges from − 0.18 MPa (Chenin blanc) to -0.05 MPa (Durif), indicating that Ψ_pd_ is typically lower than Ψ_soil_ (Table 2). The intercept is significantly different from zero for all but three of the cultivars shown, and some (Durif, Melon, Mourvèdre) not being significantly different is likely due to the slope of the regression line being very high for those cultivars. The average slope of the regression line is 1.08 (SD 0.35), ranges from 0.37 (Muscat blanc) to 1.85 (Durif) and is strongly affected by the low Ψ_pd_ values measured under dry soil conditions.

**Table 2:** Intercept and slope values of the linear regression model (LM) of Ψ_pd_ to Ψ_soil_ for 30 wine grape cultivars and 2 years of data; all slopes are significantly different (*p* < 0.001), and the p values of the intercepts are reported. The R^2^ of multiple linear regression models (MLM) for Ψ_pd_ as a function of Ψ_soil_ and vapor pressure deficit (VPD) is shown for all cultivars; the effect of Ψ_soil_ is significant for all cultivars (*p* < 0.001) and the *p* values for the VPD effect are listed. The r^2^ for the LM of the Ψ_soil_ to Ψ_pd_ difference (Ψ_pd_ − Ψ_soil_) with VPD is reported with the *p* value for VPD as the independent variable

	LM Ψ_pd_ and Ψ_soil_			MLM VPD and Ψ_soil_		LM VPD and (Ψ_pd_ − Ψ_soil_)	
	intercept	slope	*p* (intercept)	R^2^	*p* (VPD)	r^2^	*p* (VPD)
Aligoté	-0.12	1.08	< 0.001	0.62	0.77	0.01	0.60
Albariño	-0.13	0.90	< 0.001	0.75	0.01	0.10	0.04
Auxerrois	-0.16	0.91	< 0.001	0.79	0.72	0.00	0.90
Barbera	-0.08	1.38	0.00	0.64	0.10	0.12	0.03
Cabernet franc	-0.14	1.11	< 0.001	0.70	0.55	0.02	0.34
Cabernet Sauvignon	-0.11	1.22	< 0.001	0.77	0.00	0.26	< 0.001
Chardonnay	-0.14	0.57	< 0.001	0.81	0.00	0.01	0.62
Chenin blanc	-0.18	0.47	< 0.001	0.36	0.12	0.00	1.00
Durif	-0.05	1.85	0.14	0.71	0.75	0.08	0.07
Gewürztraminer	-0.09	0.91	< 0.001	0.74	0.94	0.00	0.70
Green Veltliner	-0.07	1.07	0.01	0.78	0.57	0.02	0.39
Grenache	-0.06	1.04	0.01	0.78	0.07	0.09	0.07
Lemberger	-0.15	1.26	0.00	0.57	0.03	0.15	0.01
Malbec	-0.08	0.88	< 0.001	0.68	0.66	0.02	0.39
Melon	-0.05	1.59	0.11	0.77	0.04	0.19	0.01
Merlot	-0.11	1.00	< 0.001	0.67	0.59	0.01	0.62
Mourvèdre	-0.05	1.41	0.08	0.67	0.02	0.21	0.00
Muscat blanc	-0.11	0.37	< 0.001	0.57	0.37	0.11	0.04
Nebbiolo	-0.12	1.04	< 0.001	0.71	0.21	0.04	0.20
Petit Verdot	-0.12	0.92	0.00	0.52	0.45	0.01	0.66
Pinot blanc	-0.13	0.99	< 0.001	0.62	0.86	0.00	0.85
Pinot gris	-0.12	1.08	0.00	0.49	0.51	0.00	0.69
Pinot noir	-0.13	0.85	< 0.001	0.57	0.31	0.00	0.74
Riesling	-0.08	1.15	0.02	0.65	0.99	0.00	0.71
Sangiovese	-0.05	1.69	0.01	0.85	0.01	0.30	< 0.001
Sauvignon blanc	-0.10	0.72	< 0.001	0.54	0.59	0.05	0.16
Sémillon	-0.12	1.19	0.00	0.57	0.90	0.00	0.76
Tempranillo	-0.13	1.01	< 0.001	0.75	0.00	0.22	0.00
Viognier	-0.06	0.96	< 0.001	0.81	0.54	0.00	0.70
Zinfandel	-0.08	1.78	0.03	0.61	0.27	0.12	0.03

If the Ψ_pd_ data are plotted as a function of the minimal Ψ_soil_ values from the retention curve data in Fig. 1 (the dashed lines below the 1:1 line on Fig. 3) the average slope of the linear regression model drops to 0.13 (SD 0.17) and the intercept increases to − 0.21 (SD 0.05) MPa. Plotting the Ψ_pd_ data as a function of the maximal Ψ_soil_ values of the retention curve data (see Fig. 1 and the dashed lines above the 1:1 line in Fig. 3) increases the average slope of the linear regression model to 1.65 (SD 0.4), but does not change the average intercept much, which increases slightly to − 0.08 (SD 0.03) MPa. If a linear regression model is applied to just the Ψ_pd_ data at θ_e_ > 0.36 (equivalent to Ψ_soil_ >  − 0.14 MPa or θ_v_ > 0.146 m^3^ m^−3^), the average intercept stays similar at − 0.11 (SD 0.03) MPa and the intercept values are significantly different from zero for all cultivars. Because some Ψ_pd_ measurements were done when the soil was extremely wet (above field capacity according to Fig. 2) many of the data points are near the origin, keeping the intercept in quite a narrow range despite the change in slope. These results confirm that in the wet soil region Ψ_pd_ is not equivalent to Ψ_soil_, instead indicating a gradient for water flow from the soil to the leaves at night. In the dry range we cannot say conclusively if the Ψ_pd_ is equal to Ψ_soil_ or if it is lower, but as most of the data fall within the possible retention curve range (blue and red lines in Fig. 3) it is probable that Ψ_pd_ does approximate Ψ_soil_ in the dry range.

The correlation of the slopes of the linear regression model between the two years per cultivar is only 0.39 (r^2^ = 0.16), which makes it more likely that the differences in slopes are due to measurement variability in the dry range than to cultivar-specific traits. The correlation of the intercepts of the linear regression model between the two years per cultivar is only 0.36 (r^2^ = 0.13), indicating that there is not a trend for cultivars from year to year. The intercepts of the linear regression model calculated for each year separately per cultivar had a positive correlation to the slopes of the same model: in 2021 the r^2^ value was 0.39, in 2022 it was 0.57. The higher correlation in 2022 was due to the Ψ_pd_ dropping to lower values at a higher Ψ_soil_ than in 2021 which made the slopes of the linear model very high in relation to the origin line; the reason for this difference between years is not known. The correlation of the intercept values of the 2021 linear regression to the intercept values calculated for the data pooled over 2 years (Table 2) was 0.92 (r^2^ = 0.85) as opposed to that of the 2022 data which was only 0.42 (r^2^ = 0.17). This discrepancy is due to a larger number of samples for the 2021 season, and the high slope values for the 2022 season data.

### Vapor pressure deficit

The effect of Ψ_soil_ and VPD on Ψ_pd_ was tested in a multiple linear regression model for each of the cultivars. The Ψ_soil_ was positively correlated to Ψ_pd_ and highly significant for all cultivars (*p* < 0.001, values not reported). The VPD was negatively correlated to Ψ_pd_ for 24 of the cultivars but was significantly correlated for only 10 cultivars (R^2^ values of the model and *p* values for the VPD are reported for each cultivar in Table 2. The effect of just VPD on the difference between Ψ_soil_ and Ψ_pd_, indicating a water potential gradient from the soil to the leaves at night, was tested in a linear regression model. The results were similar to the VPD effect in the multiple linear regression model in that VPD was negatively correlated to the difference between Ψ_soil_ and Ψ_pd_ for 21 of the cultivars but was only significantly correlated for 10 cultivars (r^2^ values of the model and the slopes with p values are reported for each cultivar in Table 2.

## Discussion

We observed variability among the soil water retention curves for our study vineyard, particularly between different locations within the vineyard (Fig. 1). Location 3 also showed large variability between soil depths. Such variability is often seen between field samples [22, 23]. For instance, Shouse et al. [22] found that the van Genuchten [24] model parameters showed a coefficient of variation ranging from 5 to 33% over an 80-m transect on a silt loam soil. Cameron [23] measured soil water retention curves for soil cores taken from one site and found that the coefficient of variation of the water content at a given suction for different depth varied from 0.3% to 14%, with the surface layers being more variable than the deeper layers. To account for this spatial variability among the soil water retention curves, we used the envelope of the retention curves (minimum and maximum) to compare Ψ_soil_ with Ψ_pd_.

This study showed conclusively for *V. vinifera* grapevines that in the wet soil range (θ_e_ > 0.36 or Ψ_soil_ >  − 0.14 MPa) the Ψ_pd_ is significantly lower than the Ψ_soil_ (average − 0.1 MPa, range − 0.05 to − 0.18 MPa, depending on cultivar), as Donovan et al. [11] found for a range of different plant species. This finding suggests there is a water potential difference from soil to leaves that drives water flow at night, which in turn indicates there is non-negligible transpiration at night. Nighttime transpiration in grapevines is reported to occur at a rate of about 10% of the daytime transpiration [25], and potential benefits of this have been postulated such as higher daytime photosynthetic rates [26], disposal of respiratory CO_2_ through incompletely closed stomata benefiting nighttime growth [27], or enhanced nutrient uptake from the soil [28].

We did not measure transpiration directly, but as VPD is the driver for transpiration it would be suspected to have a negative correlation with Ψ_pd_ or the difference between Ψ_soil_ and Ψ_pd_. However, these correlations were generally not strong, and only significant for 10 of the 30 grape cultivars evaluated here. This lack of correlation for many cultivars is supported by the finding of Dayer et al. [26] that nighttime transpiration did not respond to VPD and contradicted by others [25, 29]. Rogiers et al. [25] found an r^2^ value of 0.92 for the correlation of Ψ_pd_ to VPD for 35-year-old Sémillon vines when the θ_e_ was between 0.5 to 0.8. In that same θ_e_ range our Sémillon Ψ_pd_ as a function of VPD had an r^2^ of only 0.16, which may partially be explained by the sample size being about 20% of theirs (the results of the linear model in the θ_e_ > 0.5 range are not shown in Table 2. Furthermore, Rogiers et al. [25] found that the Ψ_pd_ of Sémillon was 20% lower than that of Cabernet Sauvignon, Chardonnay, Merlot, Pinot noir, Riesling and Sauvignon blanc grown in pots under the same wet-soil conditions. In our data, Sémillon had a larger difference between Ψ_soil_ and Ψ_pd_ (more negative intercept in Table 2) than Cabernet Sauvignon, Merlot, Riesling and Sauvignon blanc, but the difference was smaller than that of Chardonnay or Pinot noir (Table 2). Despite the Ψ_soil_ being higher than the Ψ_pd_ for all 30 cultivars studied, our data did not show consistent trends in this difference for cultivars, as evidenced by the low correlations between slopes and intercepts of the linear models from year to year. This inconsistency between years demonstrates the need to avoid making far-reaching conclusions regarding supposed differences in responses among genotypes based on data from a single growing season in the field or from small pot-grown plants.

Insufficient time in the night for the equilibrium between Ψ_soil_ and Ψ_pd_ to be established could be another cause for the difference between Ψ_soil_ and Ψ_pd_ [15, 30]. The leaf water status (i.e., Ψ_pd_ and Ψ_leaf_) and g_s_ of field-grown grapevines have been found to respond much more slowly to changing soil water availability than those of pot-grown vines [31]. Also, the Ψ_pd_ includes the apoplast osmotic potential while Ψ_soil_ does not include the osmotic potential of the soil solution (as it is calculated from the soil water content), though this is unlikely to contribute to the difference between Ψ_soil_ and Ψ_pd_ as the osmotic potential component of Ψ_pd_ is similar to that of xylem sap, which in turn is similar to the osmotic potential of the soil solution under non-saline conditions [32]. Based on soil solution and xylem sap N concentrations from Keller et al. [33] the xylem sap osmotic potential would be 0.004 to 0.014 MPa lower than that of the soil solution in vineyards where N fertilizer was applied at 0 and 100 kg ha^−2^ respectively, our vineyard was fertilized at 30 kg N ha^−1^ and had much less organic matter, so would be at the lower end of that range. Gravity would contribute 0.015 MPa to the Ψ_pd_ for a leaf cut at 1.5 m height, which is about 10% of the observed difference between Ψ_soil_ and Ψ_pd_.

We could not determine conclusively whether Ψ_pd_ is the same as or different from Ψ_soil_ in the dry soil range. The large variability on the dry side of the retention curves determined for this vineyard soil increased the uncertainty of Ψ_soil_ vs. Ψ_pd_ measurement pairs from that range, so that it is not clear if just the intercept values reported in Table 2 can be used as an indication of what the difference between Ψ_soil_ and Ψ_pd_ would be under dry soil conditions, or if that difference would change in magnitude as the soil dries. The slope of the linear model (shown per cultivar in Fig. 3, and listed in Table 2) being higher than 1 would indicate that the Ψ_soil_ to Ψ_pd_ gradient increases. This is unlikely, as it would become increasingly difficult for grapevines to extract water from drier soil at night, while the potential benefits of nighttime transpiration remain unchanged. Reduced growth associated with dryer soil would decrease the need for nighttime transpiration as a mechanism to enable respiration [27], which may allow the stomata to close more completely under drought conditions. Escalona et al. [29] found significant differences in nighttime g_s_ between grape cultivars under well-watered conditions but noted that drought stress reduced nighttime g_s_, which supports the idea that there is a Ψ_pd_ to Ψ_soil_ disequilibrium in the wet soil range and Ψ_pd_ = Ψ_soil_ in the dry range. In their work on oak trees, Bréda et al. [34] found that at θ_e_ > 0.4 the Ψ_pd_ was unaffected by θ_v_ and below that threshold the Ψ_pd_ dropped linearly with decreasing Ψ_soil_, which led them to conclude that Ψ_pd_ is a parameter that is unable to detect early stages of soil drying.

The slope of the linear model being less than 1 means that the model crosses the 1:1 line, at which point the Ψ_pd_ is higher than the Ψ_soil_. According to the EFM, under such conditions the water would flow from the plant to the soil unless the continuity of the water flow was interrupted. There are plants that have such mechanisms [35], but the grapevine does not as far as we are aware. A likely cause of this phenomenon is that part of the root zone is at a slightly higher θ_v_ than was measured by the neutron probe. For each Ψ_pd_ measurement soil water content was measured at two depths, and the higher of the two θ_v_ measurements (converted to Ψ_soil_) was used in the comparison of Ψ_soil_ and Ψ_pd_ as the Ψ_pd_ is expected to equilibrate with the wettest area of the rootzone [3]. Maertens et al. [36] showed that the soybean Ψ_pd_ in relation to two nutrient solutions at different osmotic potentials is correlated to the fraction of roots in each of the solutions multiplied by the osmotic potential. Améglio et al. [3] used this correlation to support their model prediction that Ψ_pd_ becomes stable at a level that depends on the soil and root resistances in root zones of differing Ψ_soil_, based on the assumption that the root ratios from Maertens et al. [36] are inversely proportional to the resistance ratios. They further point out that Ψ_pd_ equilibrating with the Ψ_soil_ of the wettest soil region in the root zone explains the lack of sensitivity of Ψ_pd_ to large spatial variations of soil moisture. In many cultivated crops, heterogenous soil water distribution is becoming the norm with increasingly widespread use of drip and micro irrigation. In addition, crop species such as wine grapes are often deficit-irrigated, which leads to frequent spatial and temporal fluctuations in soil moisture at different soil depths [37]. So the root density distribution could affect this relationship, but we did not collect data concerning root density distribution. However, under drip-irrigation in arid climates like the one in the present study (< 200 mm annual precipitation), grapevine roots are typically concentrated beneath the drip lines [38], where our θ_v_ measurements were taken. Schreiner et al. [39] found for a sandy soil in eastern Washington that more than 80% of fine roots were at less than 50 cm depth, and Davenport et al. [40] concluded that sampling to a depth of 45 cm and a radius of 20–40 cm from the drip emitters best reflects the amount of plant available soil water in the eastern Washington climate.

The difference in the slope of the linear model between the two years (Fig. 2B) could be due to differences in the dry-down pattern between the seasons in relation to how quickly the soil dried after an irrigation event, which was not monitored due to the low sampling frequency. As our neutron probe does not store the raw data, the effect of the calibration could not be tested. The variability in the slopes for the different cultivars in Table 2 could also be due to the variability of the soil retention parameters. The heterogeneity of the soil is what makes it difficult to quantify the soil water potential for the whole root zone and that same heterogeneity is what makes our comparison of Ψ_pd_ to Ψ_soil_ uncertain in the dry soil range. Zhang et al. [16] determined from sap-flow and gas-exchange measurements that when θ_e_ > 0.35 grapevines (Merlot) were anisohydric and below that threshold they were isohydric. The threshold is similar to the θ_e_ > 0.36 value that we found, and a difference between Ψ_soil_ and Ψ_pd_ in wet soil could be associated with anisohydric behavior, but it would be presumptuous to conclude this solely on the basis of Ψ_pd_. Although most of the Ψ_pd_ data fall between the two retention curve extremes when θ_e_ < 0.36 (Fig. 2), owing to the variability in our data, conclusively confirming or rejecting the Ψ_soil_ = Ψ_pd_ assumption for the dryer soil range (θ_e_ < 0.36) might require frequent, high-density Ψ_soil_ (as opposed to θ_v_) measurements. The disequilibrium between Ψ_pd_ and Ψ_soil_ can affect models built on the assumption that they are the same. Although normally no or little irrigation is required in many vineyards producing grapes for (red) wine production when θ_e_ > 0.36, more heavily-cropped table, raisin, and juice grape vineyards may be irrigated up to θ_e_ ≈ 0.6 [12, 41]. For the EMF our conclusion that Ψ_pd_ < Ψ_soil_ in wet soils implies that substituting Ψ_pd_ for Ψ_soil_ results in an overestimation of K in wet but not dry soil. This would subsequently overestimate the extent of the decline in K in drying soil.

## Conclusion

Our test of the Ψ_pd_ = Ψ_soil_ assumption for 30 wine grape cultivars in a vineyard with a silt loam soil in a warm and arid region showed that over 2 growing seasons the Ψ_pd_ was on average 0.1 MPa lower than Ψ_soil_ in wet soil with θ_e_ > 0.36. While our results indicate there is a water potential difference of 0.05 to 0.18 MPa for non-negligible water flow from wet soil to grapevine leaves at night, the correlation of intercept values per cultivar between years was low. Consequently, we lack the confidence to say that the difference between Ψ_pd_ and Ψ_soil_ is a cultivar specific trait, and consider it a general trait of grapevines. The variability of the retention curve data in the dry soil range makes it impossible to determine if the Ψ_pd_ is equal to or lower than the Ψ_soil_, though most of the Ψ_pd_ data do fall within the range of possible retention curves.

## Materials and methods

### Plant material and study site

The study was conducted in a 3.2-ha vineyard at the Irrigated Agriculture Research and Extension Center (46°17'N; 119°44'W; 364 m a.s.l.) in Washington, USA, during the 2021 and 2022 growing seasons. The vineyard soil is a Warden silt loam [20] and the climate in this region is characterized by very low annual precipitation of 193 mm and high potential evapotranspiration of 1040 mm (20-year average). The soil depth at the vineyard ranges from 50 to 100 cm above an impermeable caliche layer of unknown thickness. The plants originated from a collection of certified grapevines of the Clean Plant Center Northwest (Prosser, Washington, USA), and annual disease testing and rogueing (if necessary) are performed to keep the vineyard free of viral and bacterial diseases as much as possible. The vineyard has 30 own-rooted *V. vinifera* cultivars that are replicated in 3 blocks of 5 vines per cultivar randomly along the southern border of the vineyard. The vines were planted in 2010 in north–south-oriented rows on a < 2% southwest-facing slope at a spacing of 1.83 m within rows and 2.74 m between rows. The vines are double-trunked and trained to bilateral cordons 0.9 m aboveground, shoots are loosely positioned vertically by two pairs of foliage wires. Spur pruning is performed in the winter to 12 spurs with 2 buds each. A permanent volunteer species cover crop, which goes dormant during the summer, is grown between rows, and a 1.2 m herbicide strip is maintained under the vines. The vineyard is drip-irrigated with 2 L h^−1^ emitters spaced at 46 cm (4 emitters per vine) and there is a shut-off valve for each row.

### Dry-down cycles

In 2021 the cultivars were fully irrigated between budbreak and fruit set, then the soil was subjected to two dry-down cycles to create a gradual soil water deficit. All cultivars were rewatered when the first plants showed visible signs of water stress such as leaf wilting or chlorosis. The first dry-down cycle started on June 23 after fruit set, lasted 42 days and was followed by irrigation to field capacity. The second cycle began on August 17, at the onset of fruit ripening, and lasted 56 days. In 2022 the first dry-down cycle started following irrigation to field capacity at fruit set on July 5 and lasted 56 days; after rewetting the soil to field capacity only one set of Ψ_pd_ and θ_v_ measurements was done during the second dry-down cycle. Soil moisture was measured as the volumetric water content (θ_v_) at 30 and 60 cm depth using a neutron probe (HYDROPROBE Am/Be Model #503DR, Instrotek, San Francisco, California, USA) which was calibrated before each season by means of two media-filled barrels of known water content. One access tube was installed in each of the 3 replicate rows for all 30 cultivars (total of 90 access tubes) at mid-distance between two adjacent vines, which were used to measure Ψ_pd_. Soil moisture measurements were taken at -30 and -60 cm early-morning on the same day as the Ψ_pd_ measurements. The higher of the two θ_v_ measurements was the one used to compare to the Ψ_pd_, as the Ψ_pd_ is expected to correlate best to the highest Ψ_soil_ [3].

### Soil water retention curve

Intact soil cores of 135 cm^3^ were sampled in triplicate from the root zone at 15–22, 30–37 and 45–52 cm depth. The three samples were taken at equidistant locations in the vineyard (about 45 m apart), and the bulk density was calculated from the oven dry weight and the sample core volume. A pressure plate (plate no. 0675B05M1, effective pore size 0.5 μm, Soil Moisture Equipment Corp., Goleta, California, USA) and a dew point potentiometer (WP4-T, Decagon Devices, Meter Group, Pullman, Washington, USA) were used to determine a retention curve for the vineyard soil according to Bittelli and Flury [42]. The pressure plate data were collected for each sample location in triplicate at 0.01, 0.02, 0.033, 0.05, 0.1, 0.2, 0.3, and 0.4 MPa. Soil samples were disturbed and repacked into brass cylinders (5.35 cm diameter and 3 cm height) according to the average bulk density of each sample location (*n* = 3). The bottom of the cylinders was covered with a polyester mesh (250 μm opening) to hold soil in place. The soil samples were placed onto the pressure plates and saturated with 5 mM CaSO_4_ overnight. After that, the pressure plates with samples were pressurized in a pressure chamber until no outflow was observed from the chamber. Then, the samples were weighed to calculate gravimetric water content. The dew point potentiometer was calibrated with a certified 0.1 mol kg^−1^ KCl solution. The soil samples were brought to different θ_v_ by wetting the soil in disposable plastic sample dishes with deionized water and letting the water evaporate for different amounts of time. The target range of θ_v_ for the dew point measurements was that which would result in water potentials between − 0.5 and − 1.5 MPa. Each sample was measured just once, so there is no estimate of the instrument’s error range. According to the manufacturer, the WP4-T, which is a chilled-mirror device using Peltier coolers to control the sample temperature, can measure water potential to an accuracy of ± 0.1 MPa (WP4-T user manual, Decagon Devices).

The experimental water retention data were modeled by means of the van Genuchten [24] equation:

2 $${\theta }_{v}={\theta }_{r}+\frac{{\theta }_{s}-{\theta }_{r}}{{\left[1+{\left(\alpha h\right)}^{n}\right]}^{m}}$$

where, θ_v_ is the volumetric soil water content (m^3^ m^−3^), θ_s_ and θ_r_ are the saturated and residual water contents, respectively (m^3^ m^−3^), h (cm) is the pressure head (positive value used in Eq. 2), m and n are shape parameters related to the pore-size distribution ($m=1-1{n}^{-1}$) and α is related to the inverse of the air entry suction coefficient (cm^−1^). The RETC code [21] was used to fit Eq. 2 to the experimental data. The retention curve parameters were determined for each of the 9 soil samples taken, and the minimal and maximal Ψ_soil_ values for each θ_v_ were used to indicate the possible variability of the retention curve when comparing it to the Ψ_pd_ data. To determine the average retention curve parameters representative of the vineyard soil, Eq. 2 was fitted to the pooled data of all soil samples.

The θ_v_ of the soil at field capacity (θ_FC_) was taken from the pressure plate reading at Ψ_soil_ =  − 0.033 MPa. The θ_v_ at permanent wilting point (θ_PWP_) was calculated from two pairs of θ_v_ vs. Ψ_soil_ measurements from the dew point potentiometer above and below − 1.5 MPa as follows [43]:

3 $${\uptheta }_{-1.5}={\uptheta }_{1}+\left({\uptheta }_{2}-{\uptheta }_{1}\right)\frac{ln\left({\Psi }_{1}/-1.5 MPa \right)}{ln\left({\Psi }_{1}/{\Psi }_{2}\right)}$$

where the subscripts 1 and 2 indicate the values of the measurement above and below θ_-1.5_.

To facilitate comparing the reported θ_v_ values to other soil types, soil water content is sometimes presented as relative extractable soil water (θ_e_) in relation to θ_FC_ and θ_PWP_ [16]. This unitless parameter normalizes the influence of soil texture on θ_v_:

4 $${\theta }_{e}=\frac{{\theta }_{V}-{\theta }_{PWP}}{{\theta }_{FC}-{\theta }_{PWP}}$$

### *Ψ*_*pd*_* measurements*

Predawn leaf water potential (Ψ_pd_) was measured between 02:00 am and 04:00 am Pacific standard time using a pressure chamber (model 615D, PMS Instrument Company, Albany, Oregon, USA). In 2021 measurements were conducted on day 0, 14, 28, and 42 after the start of the first dry-down cycle, and on day 0, 14, 35 and 56 after the start of the second dry-down cycle. In 2022 measurements were conducted on day 0, 14, 28, 42 and 56 after the start of the dry-down cycle, and once immediately after rewatering to field capacity. From each row (*n* = 3), one healthy, fully expanded, and mature leaf between the 7^th^ and 15^th^ node was chosen from a randomly selected shoot on the vine located next to the soil moisture access tube. The leaf was gently wrapped in a clear plastic bag and the petiole was cut using a razor blade. The bagged leaf was immediately inserted in the pressure chamber and pressure was slowly increased until the first drop of xylem sap appeared on the cut surface, after which the pressure of the chamber was recorded [44].

### Weather data and VPD

Weather data at 15 min intervals were obtained from an AgWeatherNet weather station located in the vineyard [45]. The VPD (kPa) was calculated by the Tetens equation [46]:

5 $$VPD=0.61078 \mathrm{exp}\left(\frac{17.27 T}{T+237.3}\right)\left(1-RH\right)$$

where T is the temperature in °C and RH is relative humidity as a fraction.

### Data analysis

The significance of differences between Ψ_soil_ calculated for 50 θ_v_ values by means of Eq. 2 with parameters determined for each of the 9 soil samples (Table 1) was determined by ANOVA. The Ψ_pd_ = Ψ_soil_ assumption was analyzed by comparing the slope and intercept of the linear regression of the measured Ψ_pd_ data to the Ψ_soil_ data. The effect of VPD on Ψ_pd_ was tested by using VPD and Ψ_soil_ as independent variables in a multiple linear regression model for each of the 30 cultivars, where *p* < 0.05 was considered significant.

## Data Availability

The datasets used and/or analysed during the current study are available from the corresponding author on reasonable request.

## Abbreviations

EFM: Evaporative flux model
g_s_: Stomatal conductance
K: Whole-plant hydraulic conductance
VPD: Vapor pressure deficit (kPa)
θ_e_: Relative extractable soil water (unitless)
θ_r_, θ_s_, θ_v_: Residual, saturated, and volumetric soil water content (m^3^ m^−^^3^), respectively
Ψ_leaf_, Ψ_soil_, Ψ_pd_: Water potential of the leaf, soil, and leaf at predawn (MPa), respectively
